# A Deep Learning Method Approach for Sleep Stage Classification with EEG Spectrogram

**DOI:** 10.3390/ijerph19106322

**Published:** 2022-05-23

**Authors:** Chengfan Li, Yueyu Qi, Xuehai Ding, Junjuan Zhao, Tian Sang, Matthew Lee

**Affiliations:** 1 School of Computer Engineering and Science, Shanghai University, Shanghai 200444, China; lchf@shu.edu.cn (C.L.); qi_yue_yu@shu.edu.cn (Y.Q.); junjuanzhao@shu.edu.cn (J.Z.); sangt@shu.edu.cn (T.S.); 2 12th Grade, The Bishop’s School, La Jolla, CA 92037, USA; matthewbeaverlee@gmail.com

**Keywords:** sleep stage classification, electroencephalogram spectrogram, deep learning, convolutional neural network, long short-term memory network

## Abstract

The classification of sleep stages is an important process. However, this process is time-consuming, subjective, and error-prone. Many automated classification methods use electroencephalogram (EEG) signals for classification. These methods do not classify well enough and perform poorly in the N1 due to unbalanced data. In this paper, we propose a sleep stage classification method using EEG spectrogram. We have designed a deep learning model called EEGSNet based on multi-layer convolutional neural networks (CNNs) to extract time and frequency features from the EEG spectrogram, and two-layer bi-directional long short-term memory networks (Bi-LSTMs) to learn the transition rules between features from adjacent epochs and to perform the classification of sleep stages. In addition, to improve the generalization ability of the model, we have used Gaussian error linear units (GELUs) as the activation function of CNN. The proposed method was evaluated by four public databases, the Sleep-EDFX-8, Sleep-EDFX-20, Sleep-EDFX-78, and SHHS. The accuracy of the method is 94.17%, 86.82%, 83.02% and 85.12%, respectively, for the four datasets, the MF1 is 87.78%, 81.57%, 77.26% and 78.54%, respectively, and the Kappa is 0.91, 0.82, 0.77 and 0.79, respectively. In addition, our proposed method achieved better classification results on N1, with an F1-score of 70.16%, 52.41%, 50.03% and 47.26% for the four datasets.

## 1. Introduction

**Figure 1:**
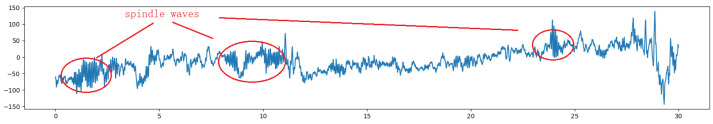
Three spindle waves of N2.

Sleep is a basic physiological need that allows our body and mind to recharge. Good sleep also helps our body stay healthy and stave off diseases. On average, a person spends one-third of their life sleeping. It is extremely important to have good sleep. Sleep specialists assess the quality of a person’s sleep by analyzing the sleep structure, which is classified into different sleep stages. Since our brain remains active during sleep, we can observe the activity state of the brain by recording electroencephalogram (EEG) signals. In practice, sleep specialists use polysomnogram (PSG) to record EEG, electrocardiogram (ECG), electrooculogram (EOG), electromyography (EMG) [1], and other physiological signals. Polysomnography is divided into 30-s sampling epochs, each of which is classified into a sleep stage per defined physiological measures. Adult sleep stages are generally divided into nighttime wakefulness (Wake), rapid-eye movement (REM) and non-REM (NREM). The Rechtschaffen and Kales (R&K) rule, an old standard proposed in 1968, divides the NREM into four stages, S1–S4 [2]. The American Academy of Sleep Medicine (AASM) as a new standard [3] integrates S3 and S4 into one stage N3. Therefore, the NREM is divided into N1–N3. Waveform and frequency are important in EEG sleep staging analysis. Experts determine the sleep stage by observing the specific waveform of each epoch. If the α (8–13 Hz) duration exceeds 50% of one epoch, it will be determined as Wake, the low-amplitude mixed-frequency wave (LAMF, 4–7 Hz) exceeds 50% of an epoch, it will be determined as N1, and the spindle wave with frequency of 11–16 Hz (most commonly 12–14 Hz) is the characteristic wave of N2, as shown in Figure 1. Observing and determining the sleep stages through the traditional method by sleep specialists is a complex process that requires expert knowledge, and tends to be tedious, time consuming and prone to error.

To reduce misclassification and time, many researchers have been trying to develop new methods or systems to automate the process. These systems can be divided into two categories: traditional machine learning and deep learning. The traditional machine learning method extracts artificial features of the signals and then uses the classic classification algorithms to classify the artificial features to obtain the sleep stages. These features usually bear clear physical significant impacts, such as sample entropy of signal, maximum signal amplitude, signal energy, etc. Deep learning, an advanced machine learning, attempts to use multi-layer neural networks to imitate the thinking of the human brain. With the increase of the number of layers, the network can extract high-level features for prediction. Compared with traditional machine learning, deep learning can greatly eliminate the problem of “feature engineering”.

Some researchers have used discrete wavelet transform to decompose EEG signals into different bands [4], and have calculated the characteristics of different bands, such as mean, entropy [5], and power spectral density (PSD) [6], which are classified based on random forest algorithms [7]. Others have used other signals, such as EOG and ECG. First, EEG and EOG are decomposed by wavelet to generate multi-bands from which various artificial features are extracted. Then, support vector machine (SVM) and other methods are used for classification [8].

Recently, deep learning has made major breakthroughs in its classification capability. The most widely used sleep stage classification method is to use EEG as an input, and then employ convolutional neural network (CNN) to extract features. Yang et al. [9] proposed a classification algorithm based on reinforcement learning to determine the model by designing the search space, optimizing the hyperparameters related to the structure and kernel, and fine-tuning the number and size of kernels in each volume layer by using a particle swarm optimization algorithm (PSO). This method simplified the training and reduced the training time of the model. Many researchers used multi-scale CNNs to extract features in order to improve the feature extraction capability of their models. These different scales of CNNs are able to focus on and extract feature information such as specific waveforms [10,11] and frequencies [12] of the signal. Good feature extraction ability of CNN allows these methods to obtain better classification performance. However, using only CNN will lead to missing and ignoring the transition relationship between sleep stages. Many researchers employ a variety of methods to learn the transition relationship of sleep stages. Mousavi et al. [13] proposed a new sleep stage classification model, called SleepEEGNet, which used a CNN to extract the features of the EEG signal and the attention mechanism to learn the transition relationship between sleep stages, which resulted in better performance for classification. Zhu et al. [14] proposed a deep learning method combining CNN and the attention mechanism for automatic sleep staging. It used CNN to learn the localization features of signals and attention mechanism to learn the feature transition relationship between epochs. Huang et al. [15] proposed a new sleep staging method that first used an attention module with improvements to make the initial classification, and then used the hidden Markov model (HMM) to learn sleep transition rules to form the final classification. This method not only achieved good results in overall classification, but also improved the accuracy of N1. Recently, long short-term memory network (LSTM) is often used to learn the transition relationship of sequence data [16]. After employing DBN to extract features, Yulita et al. [17] used one layer Bi-LSTM to learn the transition relationship of sleep stages, which improved the accuracy of sleep staging. Akara et al. [18] proposed a deep learning model, DeepSleepNet, which used a combination of CNN and two-layer bi-directional LSTMs (Bi-LSTM) to achieve automatic classification of sleep stages. Seo et al. [19] proposed the intra- and inter-epoch temporal context network (IITNet), which used jump connections for feature extraction, so that the model could learn the features related to sleep from the original EEG. It also used Bi-LSTMs to analyze the temporal relationship between sleep epoch features to complete the classification of sleep stages. Khalili et al. [20] used a data enhancement technique assisted CNN to extract features from the original EEG, then the temporal CNN to extract temporal features, and finally a conditional random field (CRF) optimization model to improve the classification accuracy of sleep staging. The results of these studies show that considering the dependence between sleep epochs can improve the effect of sleep staging. However, the attention mechanism has high model complexity. HMM and CRF can only prospectively learn the transition relationship between sleep stages.

There are some limitations in using machine learning to learn sleep stages. Firstly, researchers need knowledge of signal- and medical-related domains when extracting features, and it is difficult for machine learning to learn the transition relationship between sleep stages. Deep learning methods can learn the transition relationship between sleep stages, directly carry out end-to-end classification, and avoid the feature extraction of EEG signals. However, EEG signals do not directly reflect the information of specific characteristic waves, the above-mentioned deep learning method has an insufficient effect on the extraction of EEG frequency information, leading to poor classification performance, especially N1.

**Figure 2:**
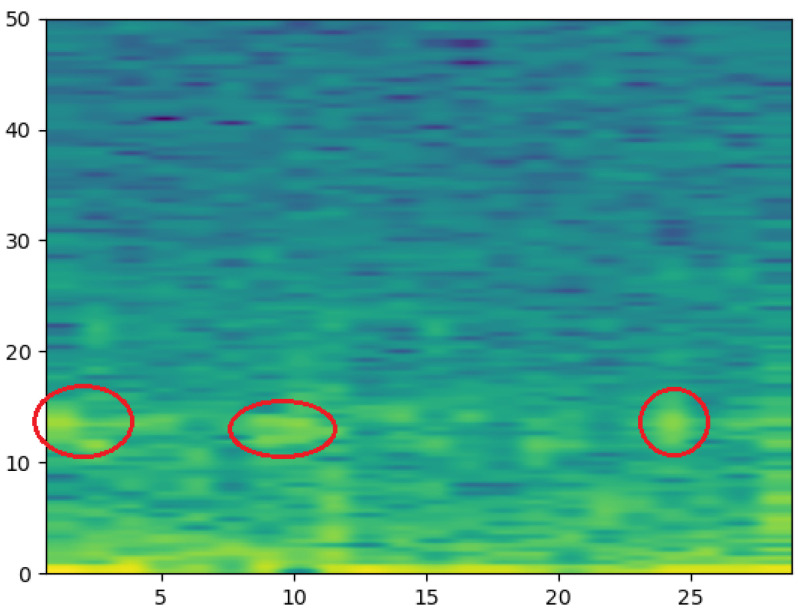
Spectrogram of N2.

**Figure 3:**
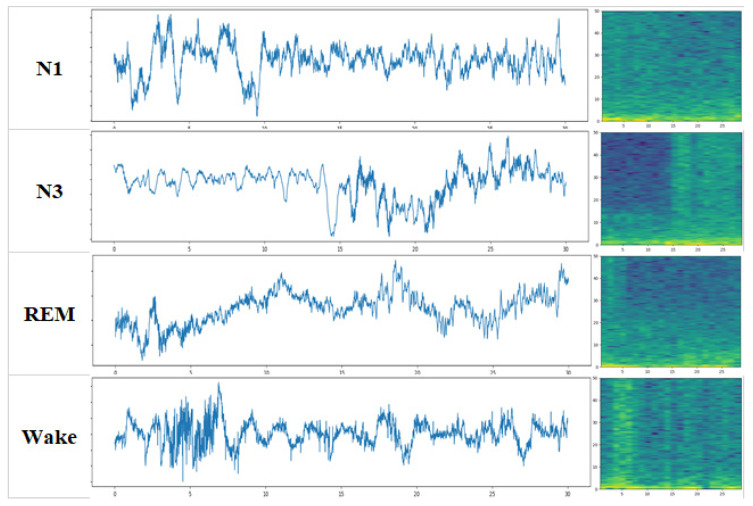
EEG and corresponding spectrograms of N1, N3, REM and Wake, respectively.

Spectrograms can reflect the activity of a specific frequency. When the EEG signal is stronger, the color of its corresponding spectrogram is brighter (yellow), and the weaker the signal is, the darker the color (blue), which is difficult to perform with EEG signals. The three spindle waves in the EEG image in Figure 1 appear in distinct yellow near 13 Hz in the corresponding time period of the spectrogram, as shown in Figure 2. Figure 3 shows the EEGs and corresponding spectrograms of the other sleep stages.

To address the above problems, we propose a new sleep staging method in this paper. The method consists of a deep learning-based model called EEGSNet. The model uses the EEG spectrogram for automatic sleep stage classification. Firstly, the CNN network with multi-layer convolution kernel is used to extract the color, position and contour information of the spectrogram, and then Bi-LSTM is used to simulate the transition relationship during sleep to improve the N1 and overall classification performance. The main contributions of this paper are summarized as follows:

1. An end-to-end classification method that uses only the spectrogram of a single channel EEG;
2. Method yields superior overall accuracy in the unbalanced data, and good results for N1;
3. The input size of the model used is fixed and there is no need to change the structure of the model or any of its parameters when a new set is used.

## 2. Materials and Methods

### 2.1. Datasets

In total, three datasets—the public benchmark databases Sleep-EDF Sleep-EDFX and SHHS were used to evaluate our proposed method.

#### 2.1.1. Sleep-EDF and Sleep-EDFX

The Sleep-EDF [21], and Sleep-EDFX [22] are two public datasets of PhysioBank. Sleep-EDF dataset contains PSG records of 8 subjects. Sleep-EDFX is an extended version of Sleep-EDF, which has 197 PSG records after two extensions in 2013 and March 2018. In both datasets, files with names starting with SC denote healthy subjects and files with names starting with ST denote subjects with mild difficulty in falling asleep. Each record in both datasets contains EEG (Pz-Oz, Fpz-Cz) and EOG signals, where the EEG signals are sampled at 100 Hz. Experts labeled nighttime sleep into six different categories based on the R&K standard for every 30-s sleep epoch: Wake, S1–S4, and REM. In this study, we used all 8 records from Sleep-EDF as the Sleep-EDFX-8 dataset and selected a total of 39 data records from 20 subjects from Sleep-EDFX as the Sleep-EDFX-20 dataset. Then, we selected a total of 153 data records from all 78 healthy subjects as the Sleep-EDFX-78 dataset. Subjects were selected according to [12]. We extracted the Fpz-Cz for each subject, segmented each record into 30-s sleep stages according to the annotation file. Based on the AASM rule, S1 and S2 were recorded as N1 and N2. Then, S3 and S4 were merged into one sleep stage N3. The first three rows of Table 1 show the number of sleep stages in the three datasets after processing.

#### 2.1.2. SHHS

The SHHS [23] dataset is a multi-center cohort study implemented by the National Heart Lung and Blood Institute to determine the cardiovascular impacts and other consequences of sleep-disordered breathing. The SHHS data contain 6441 records of men and women aged 40 years and older with each record containing EEG (C3-A2, C4-A1) and EOG signals, where the EEG signals are sampled at 125 Hz. To maintain consistency of the experimental data, we selected 329 datasets following the method of [12] and extracted the C4-A1 for each subject. The segmentation and sleep staging labeling of the signals were consistent with Sleep-EDFX. The last row of Table 1 shows the number of SHHS after processing.

### 2.2. Methods

**Figure 4:**
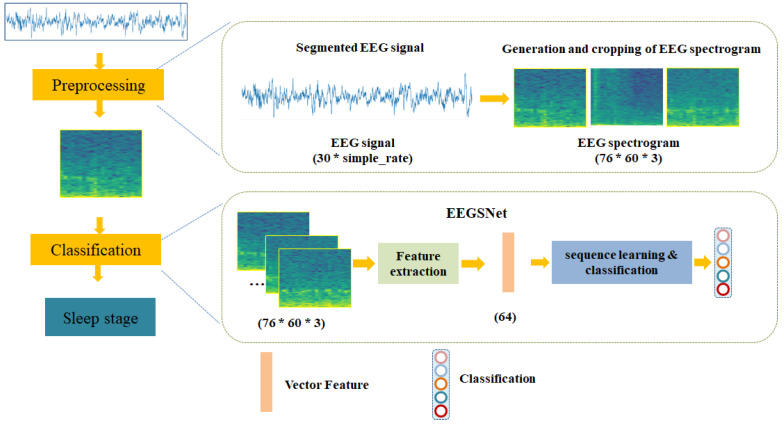
Flowchart of the proposed sleep stage classification method.

Our sleep stage classification method uses EEG spectrograms, which consists of a deep learning-based automatic sleep staging model called EEGSNet. The model takes EEG spectrogram as input. First, we preprocess the segmented EEG signal by converting the EEG into a spectrogram and cropping it. The EEG spectrogram obtained from preprocessing is applied to the sleep staging model, and then the feature extraction and serialization learning are utilized for classification. The overall structure is shown in Figure 4.

### 2.3. Preprocess

The input used by our deep learning model which we designed is the EEG spectrogram. The EEG characteristic wave of sleep stage is usually below 30 Hz [24], we set the maximum frequency of the generated spectrogram to 32 Hz. According to Nyquist theory, the sampling frequency of EEG is 64 Hz. For EEG with different sampling rates, we need to resample the data to 64 Hz.

Step 1. Resample EEG data of different frequencies to 64 Hz.

Step 2. The image size of the spectrogram is set to 1 * 0.8 inches and the dots per inch (DPI) is 100, that is, the size of the generated spectrogram is 100 * 80 dots.

Step 3. The spectrogram of EEG is obtained by Fourier transform.

Step 4. The spectrogram is cropped to keep the pixel data between the top and bottom (14, 90) and the left and right (11, 71) of the spectrogram. The purpose of this operation is to remove the irrelevant data and blank areas around the spectrogram including scale, coordinates, etc., so as to maximize the proportion of valid data in the input.

**Figure 5:**
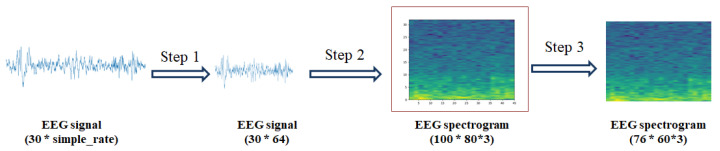
Schematic diagram of EEG generation, cropping and array transformation from signal to spectrogram.

The generation, cropping and array transformation of the EEG from signal to spectrogram are shown in Figure 5. These operations can be seen in Algorithm 1. After operations, the processed spectrogram as an image with the size of 76 * 60 * 3 is obtained as the input of sleep staging model.

**Algorithm 1** The description of EEG spectrogram produce.**Input:**
EEG segment $x\in {\mathbb{R}}^{n\times p}$ (Number of EEG segments n, Sample points per 30 s EEG segment p).
**Output:**
EEG spectrogram $y\in {\mathbb{R}}^{n\times w\times h\times c}$ (Number of EEG segments n, Weight of spectrogram w, Height of spectrogram h, Channel of EEG spectrogram c).
1: Resample EEG signal into 64 Hz
2: Set spectrogram size = (1 ∗ 0.8) inches
3: **for** i = 0; i < n **do**
4:  spctl1 = plt.specgram (
$X^{i}$) // Generate original size spectrogram.
5:  spctl2 = spctl1 [14, 11, 90, 71] // Cut spctl1. The four numbers are the starting position of the abscissa and ordinate of image, and the ending position of the abscissa and ordinate.
6: **end** for

### 2.4. EEGSNet Model

**Figure 6:**
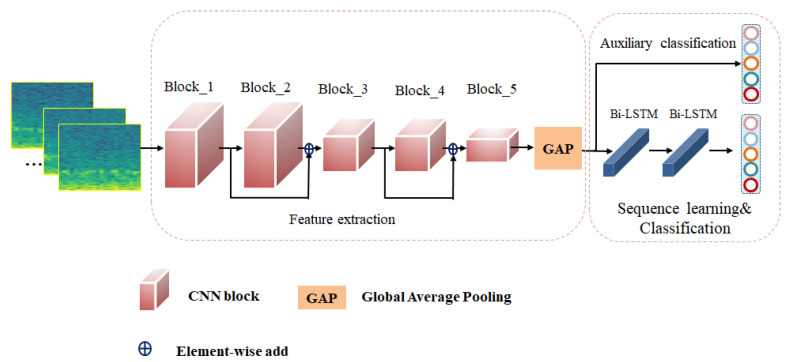
The model EEGSNet structure mainly consists of feature extraction and sequence learning. Feature extraction is where the model extracts features from the EEG spectrogram by means of 5 convolutional blocks. The features are then transferred to the sequential learning part to obtain the transition relationships between sleep stages. Auxiliary classifier is also used to help train the network.

The model EEGSNet proposed in this paper consists of two main parts: the feature extraction module and the sequence learning module. As shown in Figure 6, the feature extraction module is to extract the local features of EEG spectrogram using CNN. The sequence learning module is to use Bi-LSTM to learn the transition relationship between adjacent sleep stages and classify the sleep stages. We use an auxiliary classifier after the first part of the model’s feature extraction. The purpose of adding an auxiliary classifier is to prevent the loss of feature details during the sequence learning process.

#### 2.4.1. Feature Extraction

**Figure 7:**
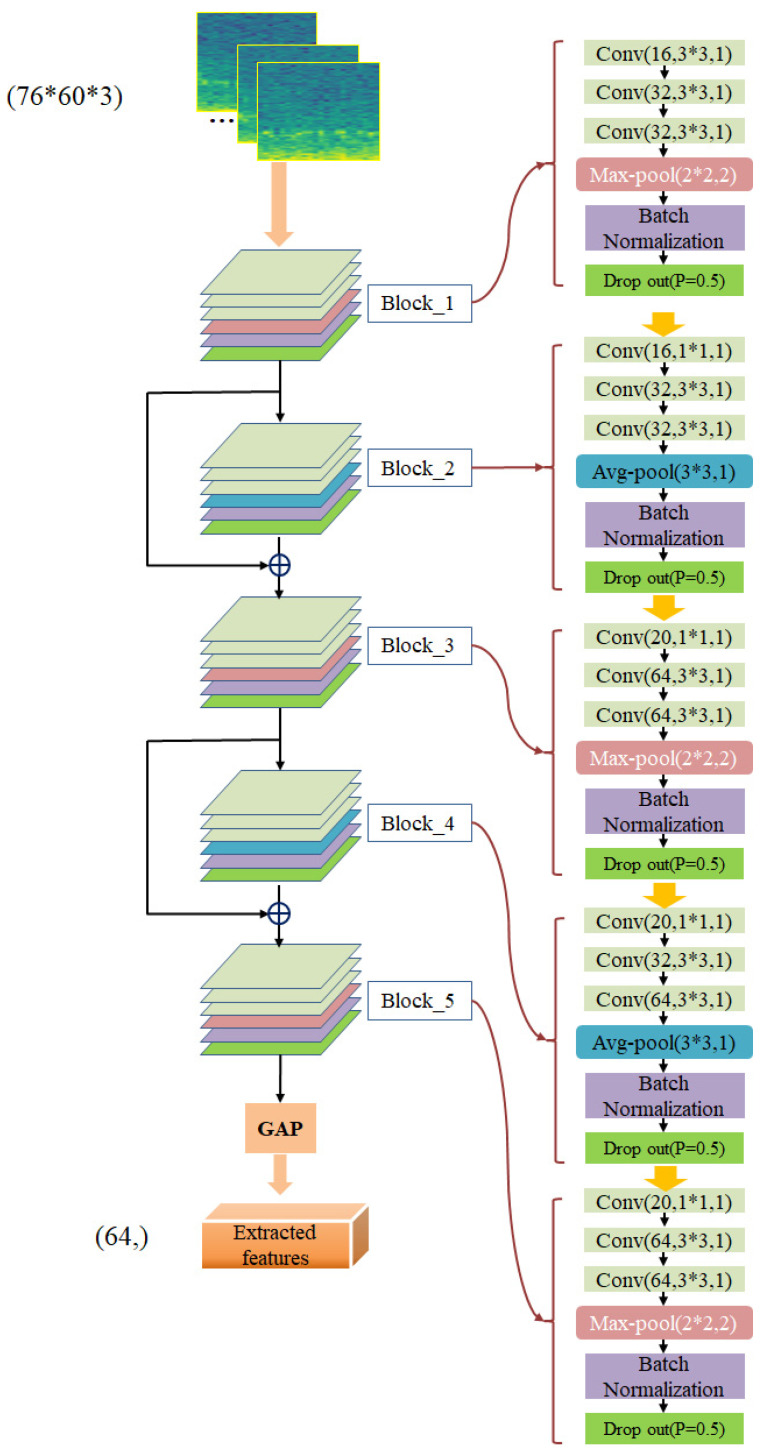
Feature extraction module, containing 5 CNN blocks, each CNN block internal hyperparameters as shown on the right.

CNN, a type of feedforward neural network, is a representative of deep learning. Different from other deep neural networks, CNNs contain a structure called convolutional kernels, and CNNs have the ability to extract local features by connecting inputs and outputs through convolutional kernels, which are suitable for models that use images as inputs. Each convolutional kernel receives relevant information from a continuous space as input and does not change the relative position of the input after convolution. The same kernel is shared by all inputs, which can effectively reduce the number of parameters. Based on this, our feature extraction module is constructed using 5 convolutional blocks and a layer of global average pooling (GAP), each convolutional block contains several convolutional layers, pooling layers, batch normalization layers, and dropout layers. The connections between the convolutional blocks are shown in Figure 7. We use two residual connections: the output of block_1 is added to the output of block_2, and the output of block_3 is added to the output of block_4. This operation allows our model to incorporate features learned from the low layers into the high layers and makes model training easy. The hyperparameters inside each convolutional block are shown on the right in Figure 7. Among them, Conv(16, 3 * 3, 1) denotes the convolution operation with kernel size of 3, the number of kernels and stride are 16 and 1, respectively. After each convolution operation, we use Gaussian error linear unit (GELU) activation function to nonlinearize the output. Max-pool(2 * 2, 2) means maximum pooling with both the kernel size and stride at 2. Avg-pool(3 * 3, 1) means the average pooling operation with kernel size of 3 and step size of 1. BN stands for the batch normalization, and Drop out(*p* = 0.5) represents the probability of retaining neural units is 0.5, which avoids the model over-reliance on certain features and can make the model more generalizable. GAP means a layer of global average pooling. After feature extraction of EEG spectrogram (76 * 60 * 3), the feature vector with the size of (64,) is obtained and output to the next module.

#### 2.4.2. Sequence Learning

**Figure 8:**
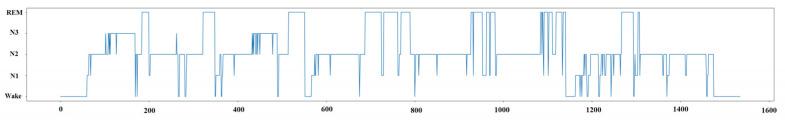
Sleep staging of SC4191E0 in Sleep-EDFX78.

The EEG of N1 is similar to that of REM, and the number of N1 is less than that of REM, N1 may be misclassified as REM [25]. However, there is a transition relationship between sleep stages, compared with N1, REM rarely appears after Wake and before N2, as shown in Figure 8. Table 2 shows the number of Wake transition to N1 and REM and transitions from N1 and REM to N2. In order to improve the classification effect of N1, our method uses the serialization model to learn this transition rule.

**Figure 9:**
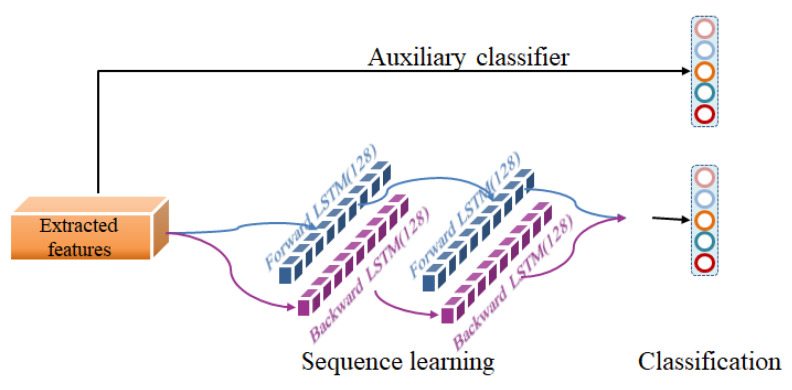
Sequence learning and classification.

Recurrent neural network (RNN) is a model of serialization learning. RNN can learn the transition relationship between sequence data. However, RNN has the problem of gradient vanish during long sequence training. As an improved version of RNN, long short-term memory network (LSTM) can solve this problem. Unlike RNN, LSTM can filter out useless information and transmit only useful information to the subsequent unit. LSTM can perform complex tasks in the longer term, but it can only obtain forward sequence information. Therefore, we use Bi-LSTM [26], a two-directional LSTM structure which can obtain both forward and backward information. The sequence length of each layer is 10, i.e., each layer contains 10 LSTM cells, and the hidden size inside each cell is 128. Figure 9 shows the connection of the auxiliary classifier and the connection details of the two-layer Bi-LSTM in the module of serialized learning. After two layers of Bi-LSTM learning, the outputs in two directions of the second layer are concatenated together for sleep staging.

### 2.5. Training Detail

We used a 20-fold cross-validation to evaluate our model. For Sleep-EDFX-8, because there were only 8 subjects, we conducted a 20-fold cross-validation of all sleep epochs (epoch-wise cross-validation). For Sleep-EDFX-20 with 20 subjects, this was leave-one-subject-out (LOSO) cross-validation. For the datasets with more than 20 subjects such as Sleep-EDFX-78 and SHHS, we divide the data into 20 groups according to subjects, and the sleep stages of each subject belongs to only one group. One group was used as the test set, and the other 19 groups were used as the training set each time. The results of each test set were combined to obtain various performance metrics. Each group of data were divided into 10 ephemeral length sequences in natural order so that the Bi-LSTM structure could learn the transition relationships of the sleep sequences. Batch size was set to 5, which meant that input 5 such sequences were entered at a time, and the number of training sessions for each group was 150. Meanwhile, we tested every 3 times using the test set to record the best evaluation results. Finally, all evaluation results were combined and averaged to obtain the overall results. We used an auxiliary classifier after the first part of feature extraction of the model. The purpose of adding the auxiliary classifier was to prevent the loss of feature details during sequence learning, and the loss weight of the auxiliary classifier was set to 0.5 and added to the final classification loss. The loss function of the model used a cross-entropy loss function, and the final loss was calculated as follows:

(1) $$Loss_{main}=-\frac{1}{N}\sum_{i=0}^{N-1}\sum_{k=0}^{C-1}y_{k}^{\left(i\right)}\log p_{k\_main}^{\left(i\right)}$$

(2) $$Loss_{aux}=-\frac{1}{N}\sum_{i=0}^{N-1}\sum_{k=0}^{C-1}y_{k}^{\left(i\right)}\log p_{k\_aux}^{\left(i\right)}$$

(3) $$Loss=Loss_{main}+0.5*Loss_{aux}$$

where *N* denoted the total number of samples; *C* denoted the number of classes; $y_{k}^{\left(i\right)}$ denoted denote the actual label of the *i*-th sample for class *k*, and $p_{k\_main}^{\left(i\right)}$ and $p_{k\_aux}^{\left(i\right)}$ denoted the predicted probability of the *i*-th sample for class *k* of main classifier and auxiliary classifier, respectively. In the entire model, the dropout layer usage probability is 0.5. It is worth noting that these auxiliary classifier and dropout layers are only used for the training phase. When using the test set, we removed the auxiliary classifier and set the dropout layer usage probability to 1.

The experiments were performed on a server with four NVIDIA Tesla V100-DGXS GPUs. The training process was implemented by utilizing Python 3.5 with TensorFlow 1.5 which is a deep learning library.

## 3. Results

### Performance

**Figure 10:**
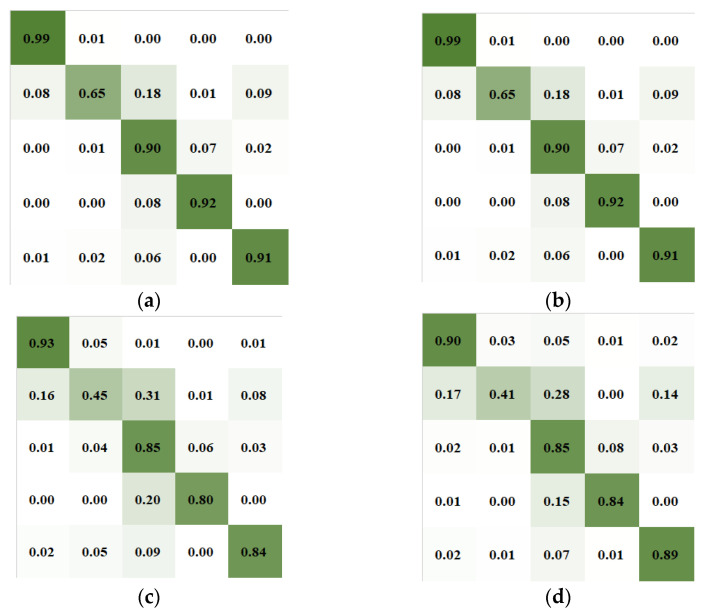
The normalized confusion matrices obtained from the four datasets in our model experiment: (**a**) Sleep-EDFX-8, (**b**) Sleep-EDFX-20, (**c**) Sleep-EDFX-78, (**d**) SHHS.

The performance of our model is evaluated by precision (PR), recall rate (RE) and F1-score (F1) at each sleep stage. Table 3, Table 4, Table 5 and Table 6 show the confusion matrices obtained by cross-validating the Sleep-EDFX-8, Sleep-EDFX-20, Sleep-EDFX-78 and SHHS datasets, respectively. Each row represents the classification of sleep stages by specialists. Each column represents the classification of sleep stages by our model. The last three columns of each row represent the performance indicators of each class calculated from the confusion matrix. The main diagonal marked in bold in each confusion matrix represents the true positive (TP) value, which represents the number of correct classifications. Figure 10 shows the result of four confusion matrices after normalization. It is clear from Figure 10 that in the classification results of the four datasets, all four sleep stages were able to obtain high classification accuracy except for N1. N1 is more likely to be incorrectly classified to the three stages of N2, REM and Wake.

**Figure 11:**
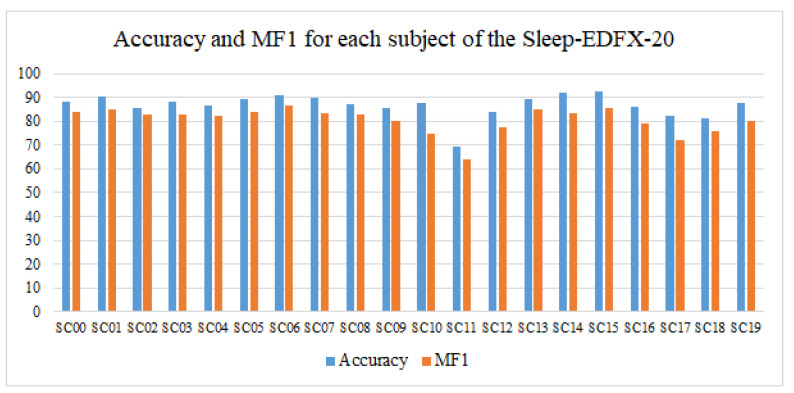
Accuracy and MF1 for each subject using 20-fold cross-validation.

**Figure 12:**
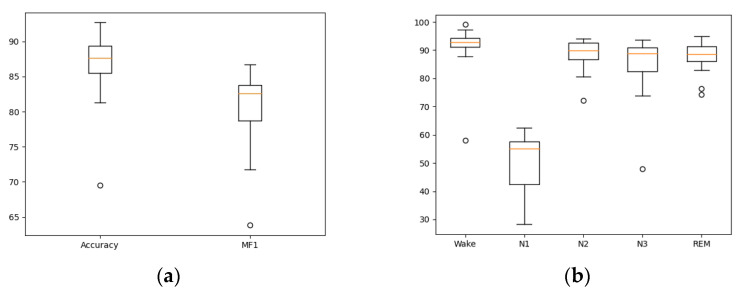
Distribution of the overall accuracy, MF1 and F1-score of each sleep stage of 20 subjects: (**a**) Accuracy and MF1, (**b**) F1 of each sleep stage.

The accuracy and macro F1-score (MF1) of our cross-validation on the Sleep-EDFX-20 dataset for each subject are shown in Figure 11. As shown in the figure, subject SC15 achieves the highest value of 92.7% in accuracy, and subject SC06 has the highest MF1 of 86.69%. Subject SC11 has the lowest accuracy of 69.53% and MF1 of 63.81%, respectively. We constructed box plots to show the overall accuracy, MF1 and F1 of each sleep stage of 20 subjects, as shown in Figure 12. From the figure, we can see that our model performs relatively consistently on the Sleep-EDFX-20 dataset.

## 4. Discussion

### 4.1. Comparison with Other Approaches

Table 7, Table 8, Table 9 and Table 10 show the comparison of cross accuracy (ACC), MF1, Kappa coefficient (Kappa) and F1-score for each class between our method and other state-of-the-art sleep staging methods on the four datasets. As seen in tables, our proposed sleep staging classification method has achieved better performance than the other methods on four datasets. Our method yields 94.17%, 86.82%, 83.02% and 85.12% classification accuracy on Sleep-EDFX-8, Sleep-EDFX-20, Sleep-EDFX-78 and SHHS datasets, respectively. They exhibit much higher accuracy than other classification methods. The MF1 and Kappa obtained by our proposed method have also outperformed the other methods. Our MF1 is 87.78%, 81.57%, 77.26% and 78.54%, respectively, and the Kappa is 0.91, 0.82, 0.77 and 0.79, respectively.

The proportion of individual sleep stages during nighttime sleep is highly unbalanced, as shown in the dataset used in this study. The number of N2 sleep stages accounted for 42.1% of the entire Sleep-EDFX-20 dataset, while the number of N1 sleep stages accounted for only 6.6% of the overall. In SHHS, the number of N2 sleep stages accounted for 43.7%, while the number of N1 sleep stages accounted for only 3%. Many classification methods usually bias the classification results towards a large proportion of classes in order to achieve a high accuracy. This may impact N1 classification. To solve the problem due to unbalanced data, [12] adds a cost-sensitive loss function to increase the weighting of small sample data in the loss function. Researchers in [20] use a data augmentation method, and [13] designs the algorithm for a two-step training process. Although these methods can improve the classification performance for small sample sleep stages, the methods can lead to a lower overall performance due to bias towards small samples. The sequence learning of our proposed method leverages the transition relationship between adjacent sleep stages and can effectively improves the classification of small samples—such as N1—without degrading the overall performance.

Table 8 also compares the number of network parameters used by EEGSNet and some deep learning methods. Compared with TinySleepNet [28], SleepEEGNet [13], XSleepNet [29] and DeepSleepNet [18], the number of parameters of EEGSNet are reduced by 0.7 M–24.1 M, which is a relatively lightweight model. Although the number of parameters of EEGSNet is 0.4 M more than that of SeqSleepNet+ [27], the number of parameters of both is the same order of magnitude, and our sleep staging method improves the classification effect, especially the F1 of N1 which is increased by 7 percentage points.

### 4.2. Ablation Experiments

To analyze the effectiveness of serialization learning in our model in improving N1 accuracy, we present an ablation research conducted on Sleep-EDFX-8 and Sleep-EDFX-20. According to the number of Bi-LSTM layers in the model, we derive the following three models:

1. EEGSNet_0: EEGSNet without Bi-LSTM.
2. EEGSNet_1: EEGSNet with one layer Bi-LSTM.
3. EEGSNet_3: EEGSNet with three layers Bi-LSTM.

**Figure 13:**
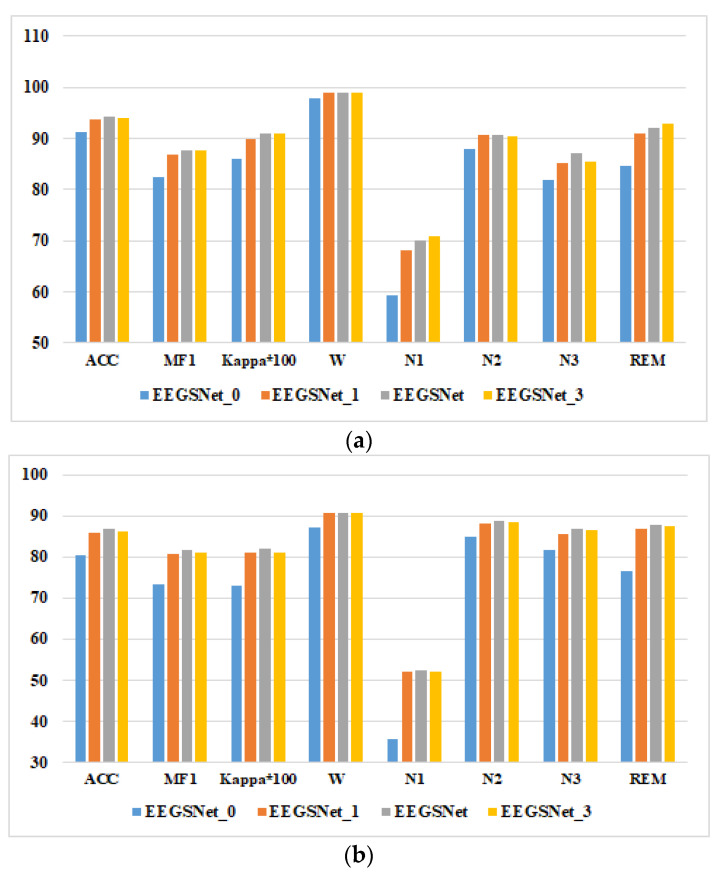
Results of ablation experiments on two datasets: (**a**) Sleep-EDFX-8, (**b**) Sleep-EDFX-20.

The results of ablation experiments on two datasets are shown in Figure 13. We can draw the following conclusions based on the ablation research. First, adding Bi-LSTM can improve the classification effect of sleep staging, especially for N1. Second, the number of layers of Bi-LSTM does not follow the concept of the more the better. With the increase of the number of layers, the complexity of the model increases. The model not only has long training time, but is also difficult to converge, resulting in network degradation. As shown in Figure 13, the classification performance of Bi-LSTM using three layers is lower than that using two layers. The classification effect is best when two layers of Bi-LSTM are used.

## 5. Conclusions

In this paper, we propose a novel EEG spectrogram-based sleep stage classification method. The method consists of a deep learning model with the ability to extract features from the EEG spectrogram and learn transition relations from adjacent epochs. Our experiments on the four datasets, Sleep-EDFX-8, Sleep-EDFX-20, Sleep-EDFX-78 and SHHS, show that our model outperforms other state-of-the-art methods, especially for N1 which tends to be more difficult to classify. In addition, we have also performed ablation experiments to demonstrate that the use of Bi-LSTM can effectively improve the classification of N1. An accurate sleep staging method can analyze sleep quality, and provide important information and support for the clinical diagnosis and follow-up treatment of sleep related diseases.

However, there are still some limitations in our research. The recognition accuracy of N1 is still much lower than that of other stages, most N1 are misclassified to N2. In addition, the introduction of Bi-LSTM leads to increased complexity of the model. In the future, we will study the feature extraction of new networks in the spectrogram and a more efficient and lightweight serialization model.

## Figures and Tables

**Table 1:** Number of subjects and sleep stage epochs in Sleep-EDFX-8, Sleep-EDFX-20, Sleep-EDFX-78 and SHHS datasets.

Dataset	Subjects	Wake	N1	N2	N3	REM	Total
Sleep-EDFX-8	8	8037	604	3621	1299	1609	15,170
Sleep-EDFX-20	20	8285	2804	17,799	5703	7717	42,308
Sleep-EDFX-78	78	65,951	21,522	69,132	13,039	25,835	195,479
SHHS	329	46,319	10,304	142,125	60,153	65,953	324,854

**Table 2:** Number of transitions from Wake to N1 and REM and transitions from N1 and REM to N2.

Dataset	Wake-N1	Wake-REM	N1–N2	REM-N2
Sleep-EDFX-8	95	0	157	14
Sleep-EDFX-20	442	32	688	136
Sleep-EDFX-78	3988	86	4269	427
SHHS	4615	623	3585	869

**Table 3:** Confusion matrix of Sleep-EDFX-8 dataset.

Stage	Predicted					Per-Class Metrics		
	Wake	N1	N2	N3	REM	PR (%)	RE (%)	F1 (%)
W	7873	46	13	7	6	99.1	99.1	99.1
N1	50	388	105	4	52	76.5	64.8	70.2
N2	8	49	3230	234	55	91.0	90.3	90.7
N3	3	0	104	1191	1	82.8	91.7	87.0
REM	14	24	96	3	1444	92.7	91.3	90.0

**Table 4:** Confusion matrix of Sleep-EDFX-20 dataset.

Stage	Predicted					Per-Class Metrics		
	Wake	N1	N2	N3	REM	PR (%)	RE (%)	F1 (%)
W	7585	291	102	20	88	89.8	93.8	91.76
N1	363	1291	673	12	429	59.8	46.6	52.41
N2	336	382	15,596	741	554	89	88.6	88.78
N3	29	1	663	4914	2	86.4	87.6	87
REM	133	194	491	0	6860	86.5	89.3	87.89

**Table 5:** Confusion matrix of Sleep-EDFX-78 dataset.

Stage	Predicted					Per-Class Metrics		
	Wake	N1	N2	N3	REM	PR (%)	RE (%)	F1 (%)
W	61,212	3406	506	44	462	93.1	93.3	93.2
N1	3359	9744	6568	133	1683	55.8	45.3	50.0
N2	539	3097	58,825	4259	2276	83.2	85.3	84.2
N3	20	22	2580	10,360	8	69.7	79.8	74.4
REM	608	1199	2263	59	21,618	83.0	84.0	83.5

**Table 6:** Confusion matrix of Sleep-EDFX-SHHS dataset.

Stage	Predicted					Per-Class Metrics		
	Wake	N1	N2	N3	REM	PR (%)	RE (%)	F1 (%)
W	41,458	1222	2261	260	900	87.2	89.9	88.6
N1	1706	4221	2857	24	1460	55.6	41.1	47.3
N2	2397	1534	120,994	11,945	4725	86.5	85.5	86.0
N3	409	2	8896	50,471	230	80.1	84.1	82.0
REM	1562	615	4824	346	58,481	88.9	88.8	88.9

**Table 7:** Comparison of performance metrics obtained by our method with other state-of-the-art algorithms on Sleep-EDFX-8.

Methods	Epochs	Overall Metrics			Per-Class F1				
		ACC	MF1	Kappa	W	N1	N2	N3	REM
Ref. [4]	15,199	90.66	76.33	0.85	97.60	31.2	88.17	84.03	80.65
Ref. [11]	15,199	91.74	82.31	0.87	98.51	54.41	89.73	86.47	82.42
Ref. [27]	15,188	93.7	84.5	0.90	98.6	52.5	**92.1**	**87.3**	91.80
EEGSNet	15,170	**94.17**	**87.78**	**0.91**	**99.08**	**70.16**	90.68	87.00	**92.00**

**Table 8:** Comparison of performance metrics obtained by our method with other state-of-the-art algorithms on Sleep-EDFX-20.

Methods	Param	Epochs	Overall Metrics			Per-Class F1				
			ACC	MF1	Kappa	W	N1	N2	N3	REM
1D-CNN-HMM [10]	-	42,308	83.98	76.9	0.78	87.8	35.1	86.6	**90.5**	86.8
Ref. [9]	-	42,309	84	75.2	0.78	89.4	30.3	88.5	89.2	78.4
Ref. [20]	-	41,950	85.39	79.27	0.8	90.5	46.6	88.4	86.1	84.6
AttnSleep [12]	-	42,308	84.4	78.1	0.79	89.7	42.6	**88.8**	90.2	79
Ref. [14]	-	42,269	82.83	77.8	0.77	90.3	47.1	86.0	82.1	83.2
IITNet [19]	-	42,308	83.6	76.5	0.77	87.1	39.2	87.7	87.7	80.9
SleepEEGNet [13]	2.6 M	42,308	84.26	79.66	0.79	89.19	52.19	86.77	85.13	85.02
DeepSleepNet [18]	24.7 M	41,950	82	76.9	0.76	84.7	46.6	85.9	84.8	82.4
SeqSleepNet+ [27]	0.2 M	42,308	85.2	78.4	0.80	90.5	45.4	88.1	86.4	81.8
TinySleepNet [28]	1.3 M	42,308	85.4	80.5	0.80	90.1	51.4	88.5	88.3	84.3
XSleepNet [29]	5.8 M	42,308	86.3	80.6	0.81	90.2	51.8	88.0	86.8	83.9
EEGSNet	0.6 M	42,308	**86.82**	**81.57**	**0.82**	**90.76**	**52.41**	88.78	87.0	**87.89**

**Table 9:** Comparison of performance metrics obtained by our method with other state-of-the-art algorithms on Sleep-EDFX-78.

Methods	Epochs	Overall Metrics			Per-Class F1				
		ACC	MF1	Kappa	W	N1	N2	N3	REM
SleepEEGNet [13]	222,479	80.03	73.55	0.73	91.72	44.05	82.49	73.45	76.06
AttnSleep [12]	195,479	81.3	75.1	0.74	92.0	42.0	**85.0**	**82.1**	74.2
Ref. [20]	191,585	82.46	76.14	0.76	92.4	48.1	84.6	73.8	81.6
EEGSNet	195,479	**83.02**	**77.26**	**0.77**	**93.19**	**50.03**	84.19	74.41	**83.48**

**Table 10:** Comparison of performance metrics obtained by our method with other state-of-the-art algorithms on SHHS.

**Methods**	**Epochs**	**Overall Metrics**			**Per-Class F1**				
		**ACC**	**MF1**	**Kappa**	**W**	**N1**	**N2**	**N3**	**REM**
DeepSleepNet [18]	324,854	81.0	73.9	0.73	85.4	40.5	82.5	79.3	81.9
SleepEEGNet [13]	324,854	73.9	68.4	0.65	81.3	34.4	73.4	75.9	77.0
ResnetLSTM [30]	324,854	83.3	69.4	0.76	85.1	9.4	86.3	87.0	79.1
multitaskCNN [31]	324,854	81.4	71.2	0.74	82.2	25.7	83.9	83.3	81.1
AttnSleep [12]	324,854	84.2	75.3	0.78	86.7	33.2	**87.1**	**87.1**	82.1
EEGSNet	324,854	**85.12**	**78.54**	**0.79**	**88.55**	**47.26**	85.99	82.03	**88.86**

## Data Availability

The data presented in this study are openly available in [Sleep Heart Health Study] at [https://doi.org/10.25822/ghy8-ks59, accessed on 10 December 2013], reference number [23], in [PhysioNet] at [https://doi.org/10.13026/C2C30J, accessed on 12 December 2002], reference number [21] and in [PhysioNet] at [https://doi.org/10.13026/C2X676, accessed on 24 October 2013], reference number [22].
